# Anomalous Diffusion and Decay of Clusters of Dopants in Lanthanide-Doped Nanocrystals

**DOI:** 10.3390/ma18040815

**Published:** 2025-02-13

**Authors:** Grzegorz Pawlik, Antoni C. Mitus

**Affiliations:** 1 Institute of Theoretical Physics, Wroclaw University of Science and Technology, Wybrzeze Wyspianskiego 27, 50-370 Wroclaw, Poland; antoni.mitus@pwr.edu.pl; 2 Social and Technical Sciences Faculty, Jan Wyzykowski University, 59-101 Polkowice, Poland

**Keywords:** upconversion in nanocrystals, Monte Carlo simulations, anomalous diffusion

## Abstract

Upconversion (UC) luminescence in doped lanthanide nanocrystals is associated with the energy migration (EM) process within clusters of dopant ions. The process of the synthesis of core–shell nanocrystals occurs at elevated temperatures, promoting the diffusion of the dopants into the shell accompanied by the decay of dopant clusters. The details of this unwanted effect are poorly understood. In this paper, we theoretically study a model of the diffusion of dopant ions in a nanocrystal using Monte Carlo (MC) simulations. We characterize the diffusion, spatial neighboring relations and clustering of dopant ions regarding the function of reduced temperature and MC time of the heating process. The dopants undergo a weak subdiffusion caused by trapping effects. The main results of this study are as follows: (i) the phase diagram of the variables reduced the temperature and MC time, which separates the enhanced and limited cluster-driven EM regimes, and (ii) a dependence of the average nearest distance between Yb ions as a function of reduced temperature, the concentration of Yb ions and MC time was found. In both cases, the requirements for an effective EM are formulated.

## 1. Introduction

The anti-Stokes emission of lanthanide UC is the process where a lanthanide ion converts the absorption of low-energy photons into a higher-energy photon emission. Because of the potential applications of this process [1], the main challenge is to increase the UC luminescence efficiency. The significant reduction in this luminescence is due to the loss of excitation energy on the nanocrystal surface. To prevent this unwanted effect, the ion should be isolated from the external environment. The most common approach to improving the UC emission is related to the addition of an undoped shell [2,3,4,5,6].

The UC luminescence of rare-earth ions depends on their structure, composition and doping concentration. The typical UC phosphors introduce Yb as a sensitizer and Er as an activator [7,8]. The simplified scheme of the UC processes in the Yb/Er system is as follows: In the first step, pumping by near-infrared light is absorbed by the Yb sensitizers. In the next step, the nearby Er ions are excited by a two-step energy transfer from the excited Yb ions. Then, subsequent nonradiative processes within the Er ions populate their radiating states. Finally, emission from the Er ions is observed, so the conversion of infrared light to visible light is achieved.

During the typical process of the synthesis of core–shell nanocrystals, the resulting mixture is heated to several hundred degrees Celsius for a few hours (for example, 300 °C for 1.5 h in Ref. [9]). The high temperature (~0.5 of melting temperature [10]) enables the diffusion of dopant ions from the core into the undoped shell. Such a process can be controlled by the time and temperature of heating. In this paper, we propose a simulation (MC) method to investigate such processes.

The presented approach constitutes a continuation and a generalization of previous numerical research [9,11,12] related to the spatial distribution of ions and the formation of the percolation paths between the Yb ions (see below). At sufficiently high concentrations, the percolation path appears. Under this condition, Yb can transfer the absorbed energy not only to Er ions but also to other neighboring Yb ions in the so-called EM process. In this paper, the previous model takes into account the diffusion of dopant ions, enabling the investigation of the temporal evolution of the system. Therefore, it is possible to analyze changes over time of the main parameters describing the spatial distribution of dopant ions. Amongst them, one of the most important is the global averaged parameter $\left\langle d_{min}\right\rangle$ [11]—the averaged value of the distance between each dopant atom and its nearest neighbor dopant atom. Another important parameter is the concentration threshold for the power law in the cluster size distribution of Yb ions [9]. In order to study the influence of Yb diffusion on the spatial range of EM between Yb ions, an analysis of the temporal evolution of Yb ions clusters will be carried out. In addition, a very important parameter of the UC in the Er-Yb system, the number of Yb ions in nearest neighborhood of the Er dopant ion, will also be studied.

To summarize, the objective of this paper is to develop Monte Carlo simulation methods for a theoretical study of the influence of temperature on the diffusion and spatial distribution of dopant ions in a core–shell nanocrystal. We investigate this phenomenon in β-NaYF4:Yb3+, an Er3+ nanocrystal structure, which was the object of a previous mixed experimental–theoretical (Monte Carlo modeling) study [9,11]. NaYF4 nanocrystals (NCs) have attracted a lot of attention in the last decade as host matrices for luminescent rare-earth (RE) ions for applications mainly in the field of optical labeling. The main argument is that, when compared with II–VI NCs (i.e., CdS and CdSe), in addition to lower toxicity, rare-earth-doped NaYF4 NCs have been additionally characterized by narrow emission bands (<10 nm) in the UV–infrared spectral range, large Stokes shifts, long emission decay times (µs or ms), weak bleaching and no blinking [13].

## 2. Materials and Methods

### 2.1. Model and Simulation Algorithm

**Figure 1:**
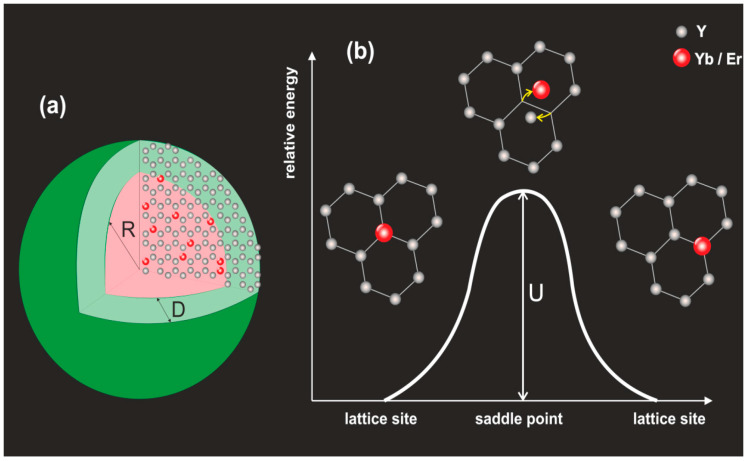
(**a**) Visualization of the geometry of the core–shell nanocrystal. D denotes the thickness of the shell. Red atoms represent ions Yb/Er in the initial state. (**b**) Potential energy diagram for the direct atom–atom exchange.

**Figure 2:**
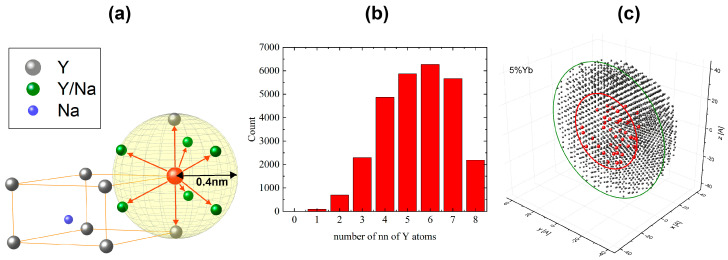
The cell for Y atoms and possible directions of movements of a dopant atom for $R_{c}=0.4$ nm (**a**). Non-normalized histogram of nearest neighbors (Y atoms) of a dopant calculated for R=8 nm (**b**). Cross-section of the nanocrystal with positions of Y atoms and 5% of dopant Yb atoms (red spheres) for the core with radius R=2.5 nm and the shell with thickness D=2 nm (**c**).

In this section, we present a very simple kinetic MC model of the migration of dopant atoms from the core into the shell of a nanocrystal, driven by a direct atom–dopant exchange of places. The model is formulated in terms of transition probabilities per unit of time. We used the standard Metropolis Monte Carlo simulation method. It consists of the following steps: Firstly, one creates a starting configuration of the system—in our case, the crystal lattice structure (β-NaYF_4_) with Er and Yb dopants. Next, a trial configuration, generated by an exchange between Yb and Y atoms, is accepted with some probability, which depends on the temperature. This procedure is repeated many times, and the trajectories of the dopant atoms are registered. The simulation programs were written by one of us (G.P.). The details of the MC simulations are as follows:

The nanocrystal is modeled as consisting of a core with radius R and a shell with thickness D. A schematic visualization of the geometry of the core–shell nanocrystal is presented in Figure 1a. In the first step of the MC simulation, the positions of Y atoms are calculated according to the crystal structure (β-NaYF_4_) in a spherical nanocrystal with diameter 2(R+D). Thus, the core and the shell have the same crystal structure. The lattice of Y atoms is partially random (Figure 2c). This feature is inherent in the β-NaYF_4_ structure. Namely, some lattice sites inside the cell of Y atoms, marked green in Figure 2a, are randomly occupied, with probability ½, either by Y or Na atoms. In this sense, the crystal structure is random and not homogeneous. Then, for a chosen concentration of dopants, assuming that their distribution is homogeneous over the entire volume of the nanocrystal’s core, the positions of dopants in the core are randomly chosen [9]. The randomness of the Y lattice implies that the local environment of a dopant becomes a random variable. In particular, each dopant atom is surrounded by one to eight nearest neighbor Y atoms in a sphere with radius $R_{c}=0.4$ nm. In Figure 2b, a non-normalized histogram of the number of nearest neighbors (Y atoms) of a dopant, calculated for R=8 nm, is shown. The positions of atoms and dopants generated by this procedure constitute the initial configuration for the kinetic MC simulation of the process of dopants’ diffusion in the random Y-atom lattice; see Figure 2c.

The model of the dynamics of the dopants neglects vacancy-driven diffusion, promoting a direct atom–dopant exchange to the main diffusion mechanism of the dopants in both parts (core and shell) of the nanocrystal [14,15]; see Figure 1b. Namely, a dopant (Er/Yb) exchanges its position with a next-neighboring Y atom as a result of an over-barrier transition. This process neglects an exchange with Y atoms at larger distances and is motivated by the fact that over-barrier transitions for those atoms are less probable. In particular, the exchange becomes effective if the distance between the atoms is smaller than $R_{c}=0.4$ nm; see Figure 2a. The transition rate *P* of an exchange between Y and dopant atoms is given by the standard kinetic formula [16]:

(1) $$P=Ae^{-\frac{U}{k_{B}T}},$$

where U denotes the energy barrier (Figure 1b), constant A is related to the vibration frequency of the migrating atoms, $k_{B}$ denotes the Boltzmann constant and T stands for the absolute temperature. The energy *U* is used to scale the temperature and to define the reduced temperature $T^{*}=k_{B}T/U$. In the MC simulations, the transition rate (with A=1) refers to a unit of MC “time”, one MC step (MCS), and *P* becomes the probability of an atom–dopant exchange. To avoid misunderstanding, let us point out that while the transition in question is purely a quantum-mechanical effect, in a classical Monte Carlo modeling the transition rate P is not directly related to quantum chemistry. So, in particular, the model does not account for relevant quantum-mechanical parameters like, e.g., differences between the radii of the atoms or different possible oxidation states of erbium and ytterbium.

**Figure 3:**
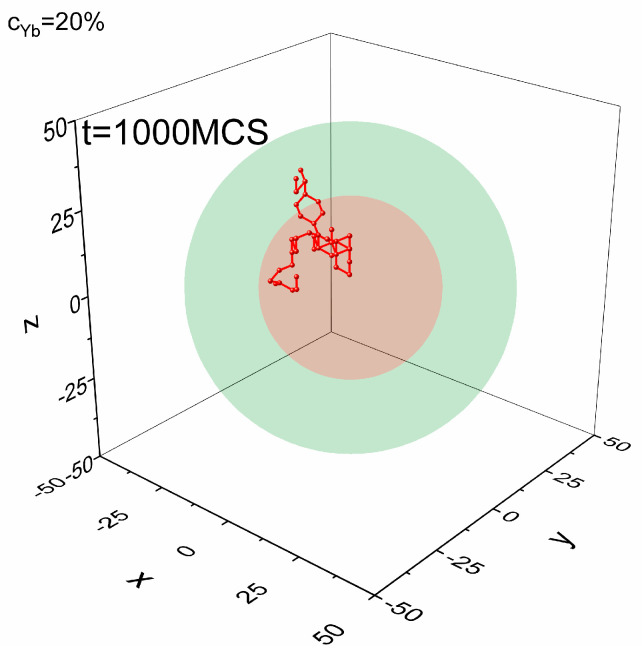
Trajectory of a chosen dopant atom in core–shell system for 1000 MCS ($T^{*}=0.4)$.

The migration of dopant atoms is modeled as follows: The dopant has, as explained above, some number of nearest-neighbor Y atoms; see Figure 2a. In a single MCS, one of them is randomly chosen. Then, a jump of the dopant atom is performed with probability P (Equation (1)). This procedure is applied to each dopant atom in the system in a single MCS. In Figure 3, a sample trajectory for a chosen dopant atom is shown in a time interval of 1000 MCS. In Monte Carlo modeling, the MCS and the constant A (Equation (1)) are related to a time unit in a real experiment.

It is important to point out that if one (or more) of next-neighbor Y atoms (green spheres in Figure 2a) are missing, then the process of the diffusion becomes asymmetric in the sense that displacements in the corresponding directions become blocked. Thus, the MC kinetics of dopants becomes a random walk with random obstacles.

### 2.2. Characterization of the Displacement of the Dopant

In the initial configuration, a dopant atom is placed close to the center of the nanocrystal. Next, it diffuses during 1000 MCS. This procedure is repeated 1000 times for each $T^{*}$. We characterize the dynamics of dopant atoms using the mean square displacement. The displacements are calculated in each MCS over a statistical ensemble consisting of $N=10^{3}$ independent trajectories: ${\left(∆\vec{r}\right)}^{2}\left(t\right)=\frac{1}{N}\sum_{i=1}^{N}{\left({∆\vec{r}}_{i}\right)}^{2}\left(t\right)$, where *t* denotes time in the units of MCS. Based on the linear fit of the log–log plot of ${\left(∆\vec{r}\right)}^{2}\left(t\right)$ against *t* (for some range of *t*), the exponent γ is calculated:

(2) $${\;\left(∆\vec{r}\right)}^{2}\left(t\right)\propto t^{\gamma }$$

Various types of diffusion—normal or anomalous—are characterized by the value of exponent γ. Gaussian (normal) diffusion corresponds to γ=1, while γ<1 and γ>1 characterize subdiffusion and superdiffusion, respectively. More details related to such an analysis can be found, e.g., in Ref. [17].

## 3. Results

### 3.1. Characterization of Anomalous Diffusion of Dopant Atoms

**Figure 4:**
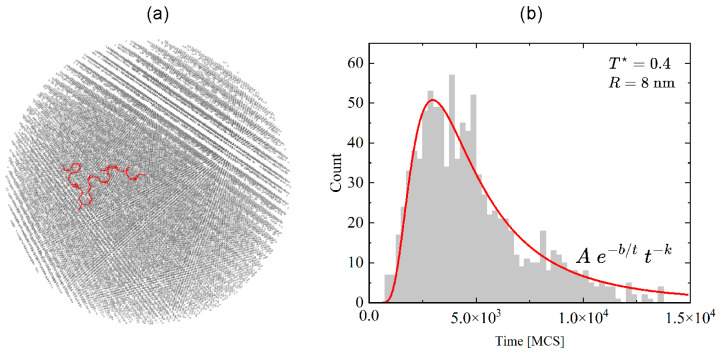
Exemplary trajectory of a dopant ion (red) in time interval of 1000 MCS and positions of 27,985 Y atoms (gray) (**a**). Unnormalized histogram of the first passage time for dopant atom to cross the surface of the nanocrystal (with the mean value $4.8\times 10^{3}$ MCS) for R=8 nm, $T^{*}\;$= 0.4 (**b**).

In this section, we analyze some general features of the diffusion of dopant atoms in a nanocrystal with radius R=8 nm at various temperatures—the core–shell geometry plays no role here. Figure 4a shows a typical trajectory of the dopant atom. A histogram of the time necessary for a dopant atom to reach the surface, the so-called first passage time (FPT), calculated for $T^{*}=0.4$, is shown in Figure 4b. Two conclusions can be drawn. Firstly, the shortest FPT is around 1000 MCS. Thus, this size of the nanocrystal allows one to safely analyze the diffusion processes up to 1000 MCS at this temperature because the dopant atoms are confined within the nanocrystal. Secondly, the MC dynamics of the dopant is not diffusive. Namely, the probability density function (pdf) for FTP in the case of the Wiener process is given by Pearson’s 5 formula [18], with the long tail proportional to $t^{-3/2},$ while in our case, it drops down as $t^{-3.9}$.

**Figure 5:**
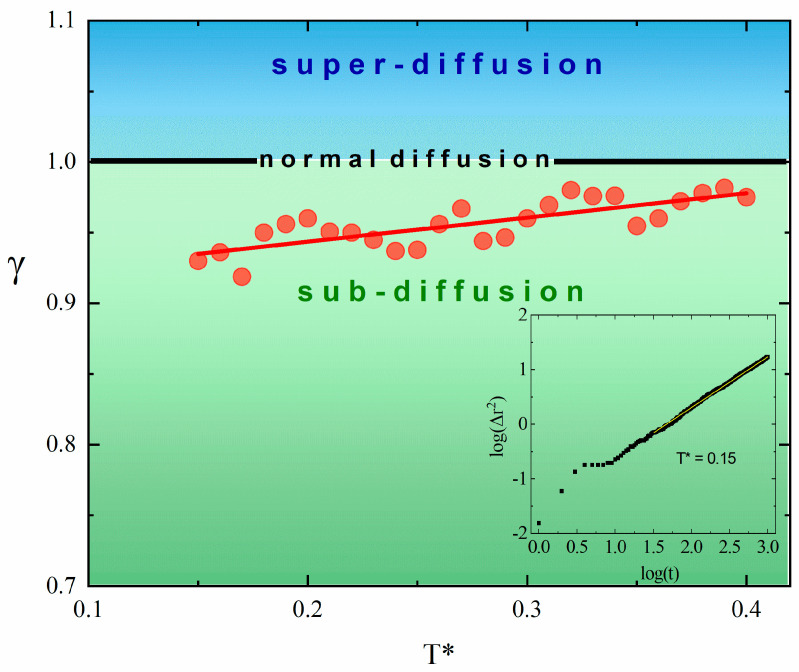
Temperature dependence of the exponent γ (red full circles) and linear fit (red line). The log–log plot of displacement ${\left(∆r\right)}^{2}\left(t\right)$ and linear fits for the time interval $1.5<log\left(t\right)<3$ for T* = 0.15 (inset).

The plot of the exponent γ as a function of the temperature is shown in Figure 5. The fitting procedures were performed in the interval of time $1.5<log\left(t\right)<3$ and used linear log–log plots like the one shown in the inset. The value of the exponent *γ* slowly increases with the increasing temperature, but remains in the regime γ<1. This corresponds to a weakly non-Gaussian subdiffusive behavior, supporting the conclusion of an analysis of pdf. We tentatively ascribe this effect to a hindered diffusion caused by the partially random lattice of Y atoms.

**Figure 6:**
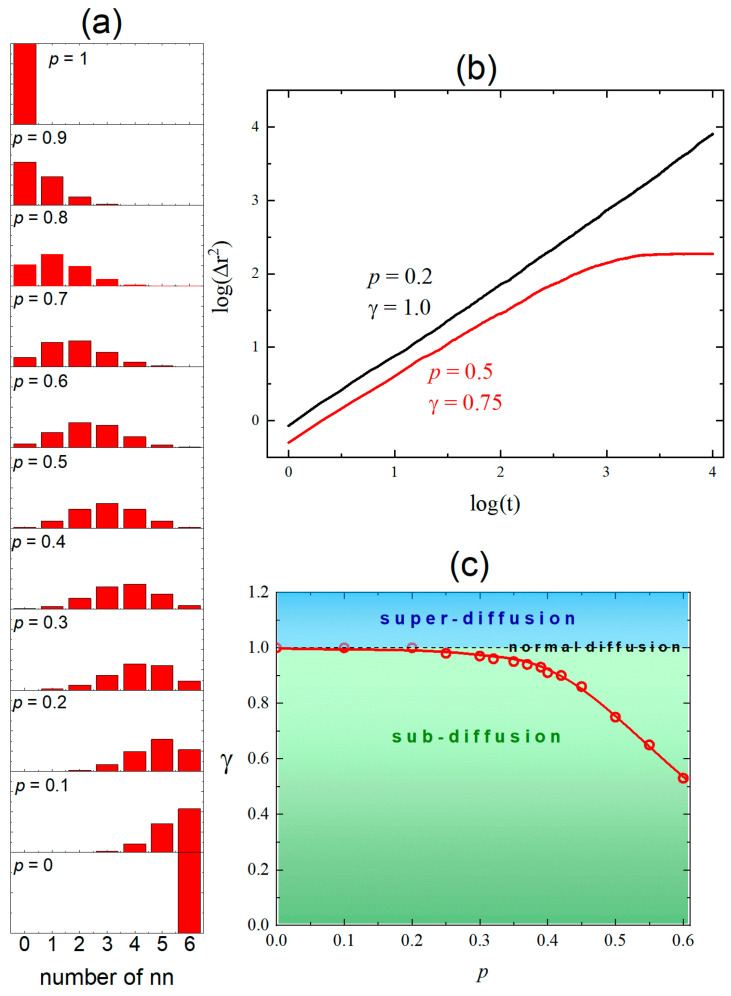
The average number of nearest neighbors in a simple cubic lattice as a function of obstacle concentration (**a**) and the crossover from normal to anomalous diffusion (**b**,**c**).

To obtain a deeper understanding of the origin of observed subdiffusion, we study a toy athermal model of an obstructed diffusion, with one free parameter. Namely, the sites of a 3D simple cubic lattice are randomly occupied with probability p by the obstacles. Each lattice site has six nearest neighbors. The diffusion of a random walker is observed during $10^{3}$ MC steps. In each step, the walker can perform a step to one of the nearest neighbor sites, provided the randomly chosen direction of movement is not marked as an obstacle. This procedure is repeated for $10^{3}$ independent systems for a given value of *p*. As the concentration *p* of the obstacles increases, the number of accessible nearest neighbors (nns) decreases (Figure 6a). The average number $N_{SC}$ of accessible nns decreases linearly with *p*: $N_{SC}=6(1-p)$. The decrease is accompanied by the crossover from normal to anomalous diffusion—in Figure 6b, low concentration (p=0.2) corresponds to normal diffusion while p=0.5 promotes a subdiffusion. Figure 6c summarizes the dependence of exponent γ on probability p. Above the threshold $p_{T}$, say around 0.25, subdiffusion is present; below, normal diffusion is observed. This study was performed for the values of probability *p* < 0.6 because the analysis of the data relies on the linear part of the plot (Figure 6b). For larger probabilities, the nonlinear behavior sets in after a short MC time, and the analysis becomes complicated. This interval of probabilities is sufficient to present the effect of the crossover between two regimes of diffusion. Similar results were obtained for 2D system in Ref. [19].

In spite of the fact that the types of randomness of the lattice are different in both cases, the toy model nicely explains the presence of a weak subdiffusion in the model nanocrystal. Namely, the average number $N_{NCS}$ of accessible nns calculated from the histogram (Figure 2b) reads $N_{NCS}=5.44$, and the maximal number of nn atoms is eight. To interpret the results for the NCs in terms of the toy model, we compare the average number of accessible nn atoms for a single atom. One finds the equation $N_{NCS}/8=N_{SC}/6$ or 0.68=1−p, and finally, p=0.32. The exponent *γ* calculated for p=0.32 reads γ(0.32)≈0.95 (Figure 6c), nicely reproducing the weak subdiffusion in NCs (Figure 5).

**Figure 7:**
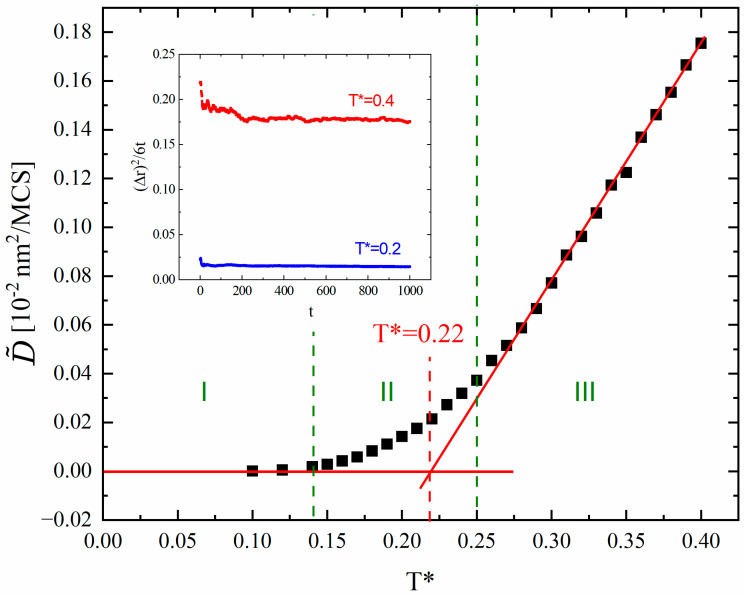
Plots of the upper boundary for the diffusion constant $\tilde{D\;}$ against temperature. Inset: time dependence of ${\left(∆\vec{r}\right)}^{2}/(6t)$.

Let us now discuss the temperature-dependent time scales for the diffusion in NCs. In three dimensions, the diffusion constant for normal diffusion is defined by the relation $D\left(T\right)=\underset{t\to \infty }{\lim}\left[{\left(∆\vec{r}\right)}^{2}/(6t)\right]$. In Figure 7 (inset), an exemplary temporal dependence of ${\left(∆\vec{r}\right)}^{2}/(6t)$ is presented. It should be pointed out that the time window contains only three decades of time. We find that $\frac{{\left(∆\vec{r}\right)}^{2}}{6t}$ is, apart from statistical fluctuations, a decreasing function. This feature is typical for a subdiffusion, described in terms of fractional diffusion equations [20,21], where the diffusion coefficient is calculated using time scaled by the exponent *γ*. One of the standard approaches in situations when long simulations are not possible (which is the case of NCs) introduces the concept of an upper boundary for the diffusion constant $\tilde{D\;}$ [22]. Namely, it is defined as the value of ${\left(∆\vec{r}\right)}^{2}/(6t)$ for the longest simulation time available, in our case *t* = 1000 MCS.

In Figure 7, a plot of the function $\tilde{D\;}$ vs. $T^{*}$ is shown. Based on this plot, three ranges of temperatures can be found: (I) low temperatures for $T^{*}<1.4$ where the diffusion mobility of the dopant ions practically does not exist, (II) the range ${1.4<T}^{*}<2.5$ with increasing values of $\tilde{D\;}$ and (III) $T^{*}>2.5$, where a linear increase is observed.

### 3.2. MC Dynamics of Dopant Atoms and Energy Migration in Core–Shell Nanocrystal

In this subsection the nanocrystal has a core with radius R=2.5 nm and a shell with thickness D=2 nm.

#### 3.2.1. Average Distance $\left\langle d_{min}\right\rangle$ Between the Dopants

**Figure 8:**
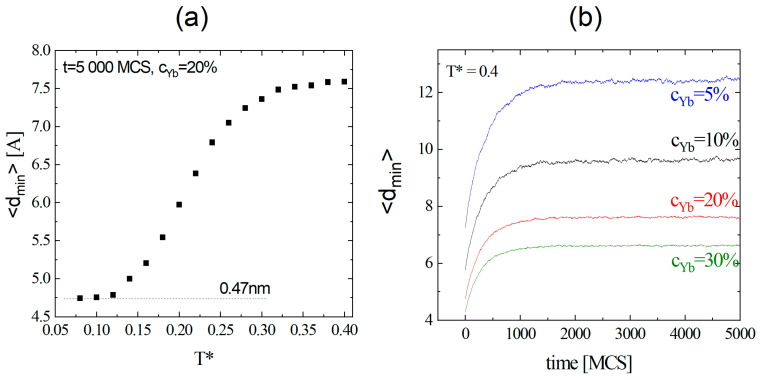
Plot of average distance $\left\langle d_{min}\right\rangle$ as a function of reduced temperature T* (**a**). Temporal dependence of $\left\langle d_{min}\right\rangle$ for a few dopant concentrations at T* = 0.4 (**b**).

The migration of dopant atoms from the core into the shell results in an increase in $\left\langle d_{min}\right\rangle$. The efficiency of migration and the increase in $\left\langle d_{min}\right\rangle$ are determined by reduced temperature $T^{*}$. For the temperatures below the threshold $T_{th}$= 0.14, $\left\langle d_{min}\right\rangle$ is independent of the temperature; above it, a strong increase in $\left\langle d_{min}\right\rangle$ is observed (Figure 8a). As expected, the threshold temperature corresponds to an onset of diffusion (Figure 7). The temporal dependence of $\left\langle d_{min}\right\rangle$ for a few concentration $c_{Yb}$ values is shown in Figure 8b. Its rapid increase in the first phase of the diffusion is followed, after 1000–3000 MCS, by a quasi-plateau where the dopants reach the surface of the nanocrystal; see Figure 4b.

**Figure 9:**
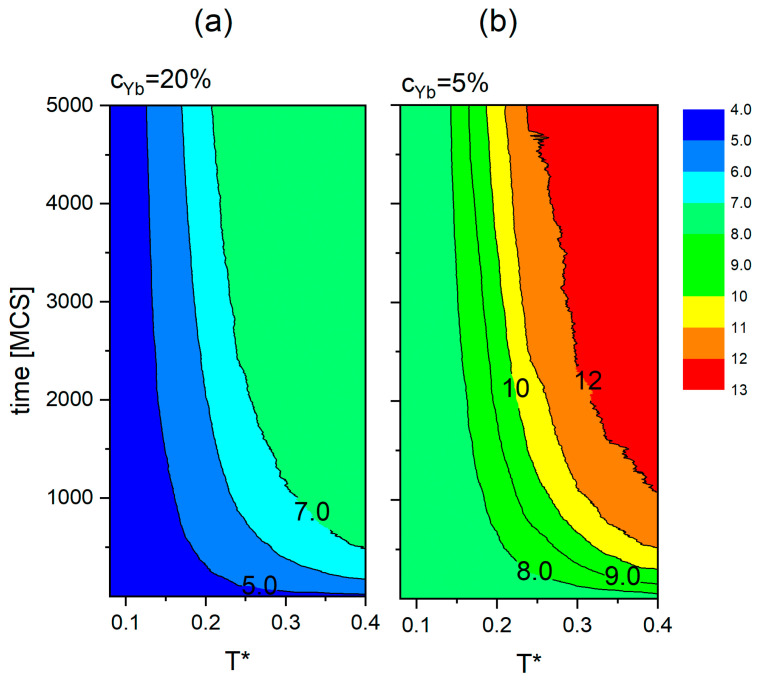
The dependence of $\left\langle d_{min}\right\rangle$ on time and temperature T* for the dopant concentration 20% (**a**) and 5% (**b**).

**Figure 10:**
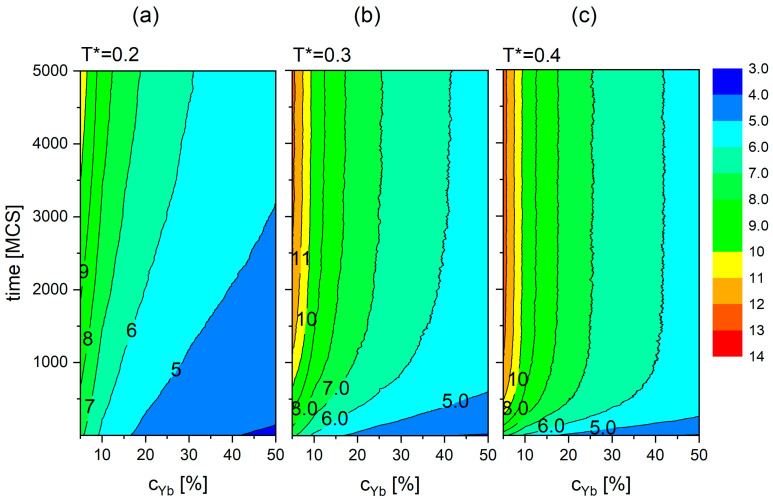
The dependence of $\left\langle d_{min}\right\rangle$ on time and dopant concentration $c_{Yb}$ for $T^{*}=0.2$ (**a**), $T^{*}=0.3$ (**b**), $T^{*}=0.4$ (**c**).

The dependence of $\left\langle d_{min}\right\rangle$ on time and temperature for the dopant concentrations equal to 5% and 20% is presented in Figure 9. After 5000 MCS elapse, for reduced temperature in the range $0.1\leq T^{*}\leq 0.4$, the values of $\left\langle d_{min}\right\rangle$ span the interval (4.7−7.7) Å for $c_{Yb}=20\%$ and (7.1−12.6) Å for $c_{Yb}=5\%$. The conclusion drawn on the basis of the two plots, namely the faster increase in <*d_min_*> with time and temperature for lower concentrations of the dopants, holds in general. A systematic study of this effect goes beyond the scope of this paper. Similar maps of $\left\langle d_{min}\right\rangle \left(t,c_{Yb}\right)$ as functions of time and concentration $c_{Yb}$ for $T^{*}=0.2$ (a), $T^{*}=0.3$ (b) and $T^{*}=0.4$ (c) are shown in Figure 10. The relationships presented on those maps (Figure 9 and Figure 10) may enable a better control of the optical properties of core–shell nanocrystals. This topic is briefly discussed in the last section.

#### 3.2.2. Clustering of Dopants and Energy Migration

**Figure 11:**
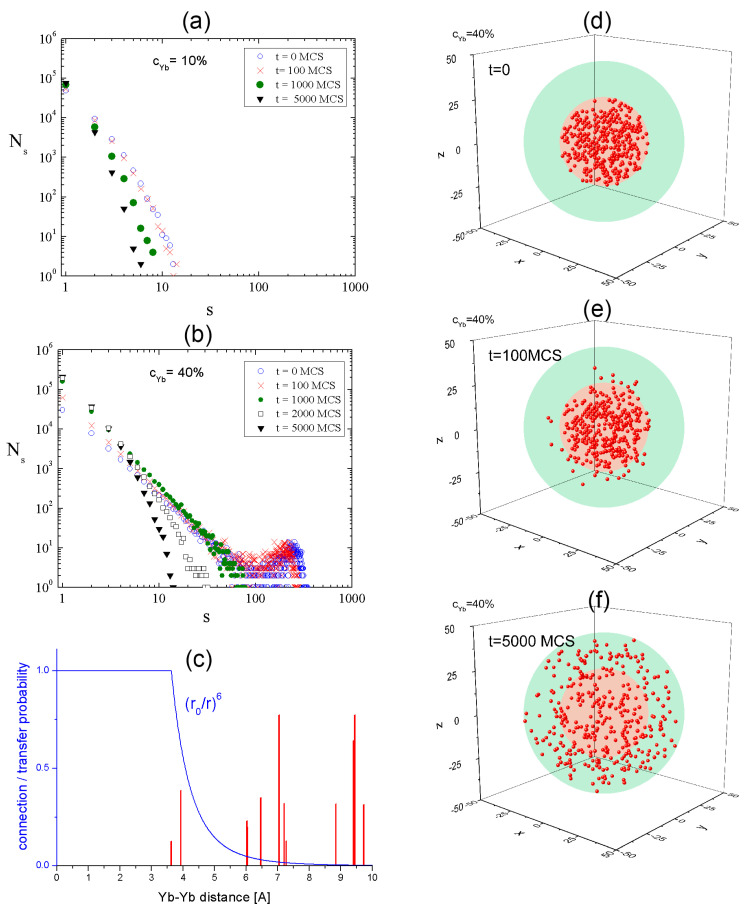
The distribution of sizes of Yb clusters for $c_{Yb}=10\%$ (**a**) and $c_{Yb}=40\%$ (**b**). Connection/transfer probability between Yb ions. Vertical lines denote nearest distances between Yb ions (**c**). Temporal dependence of spatial distribution of Yb ions (**d**–**f**). Calculations were performed for $T^{*}=0.3$.

EM was characterized as a percolation-type migration in a cluster of Yb ions [1]. In order to study the influence of Yb diffusion on the characteristic scale of length of EM between Yb ions, we analyze the temporal evolution of the clusters of Yb ions. The definition of an Yb cluster is based on the concept of connection/energy transfer probability for two Yb atoms. Namely, when this probability is sufficiently large, the two atoms belong to a cluster. To quantify this criterion, we use the theory of Förster [23], which predicts a strong decrease in the probability, proportional to $r^{-6}$, for r larger than $r_{0}=3.6\;Å$ (r denotes the distance between the two Yb atoms); see Figure 11c. The vertical lines in Figure 11c denote the distances between nearest Yb ions. One finds that the energy transfer probability is non-negligible when the distances between two Yb ions are smaller than 4 A. Thus, only such ions can form a cluster. In problems related to the percolation [24], the distribution of cluster sizes plays the central role. In our case, it depends on the concentration of the dopants. Figure 11a,b show those distributions in the core, for $c_{Yb}=10\%$ and $c_{Yb}=40\%$, at $T^{*}=0.3$. In the first case, there are, on average, 85 Yb ions in an NC. The largest cluster consists of 14 ions in the first stage of diffusion and only 6 ions after 5000 MC; hence, EM between Yb ions is strongly limited. On the other hand, for a sufficiently large concentration, $c_{Yb}=40\%$, the temporal evolution of the cluster size is significantly different. The average number of Yb ions is about 342. The largest cluster in the initial state (*t* = 0 MCS) consists of 324 Yb ions (95% of all Yb ions in the system). For the time t<1000 MCS, clusters with the size of s≥200 frequently appear in the system. The times close to t=τ=1000 MCS are very specific: a linear distribution of cluster sizes in log–log scale is present (Figure 11b), with the largest cluster around the size of 80 (23% of all Yb ions). For t>τ=1000 MCS, big clusters are not observed, and the distribution of the sizes of the clusters becomes similar to that for the small concentration.

The time τ introduces an important temporal scale, referred to as a critical time threshold. For long times, t>τ, small clusters are dominant in the system, and EM between Yb ions mediated by clusters is strongly limited. For short times, t<τ, cluster-mediated EM has a large spatial range and may play an important role in the optical properties of the system. The critical threshold time corresponds to a starting point of the decay process of big clusters, promoting smaller ones. As expected, the critical time threshold corresponds to an onset of the diffusion of Yb ions from the core to the shell; see Figure 11d–f.

The critical threshold time depends on temperature T* and concentration $c_{Yb}$:

(3) $${\tau =\tau (T^{*},c}_{Yb}).$$

**Figure 12:**
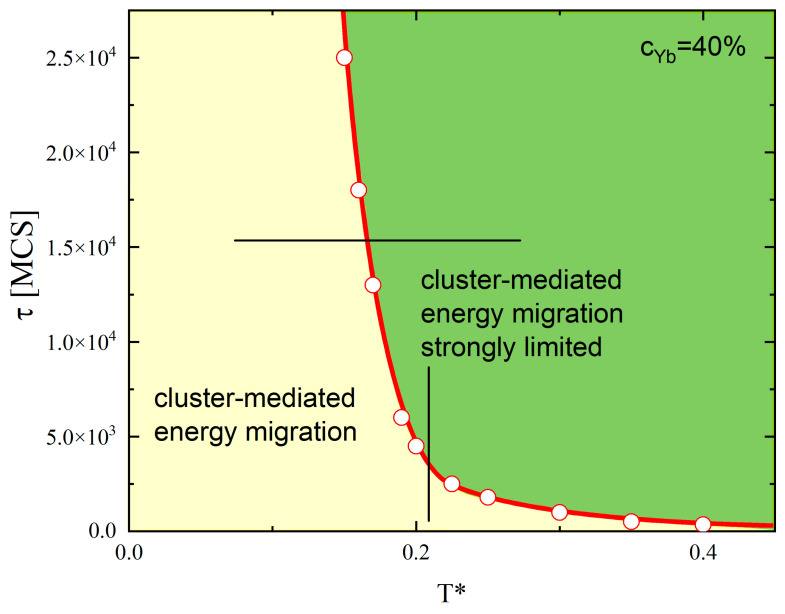
The dependence of the threshold time τ on reduced temperature; see text for more details.

Figure 12 shows the plot of ${\tau (T^{*},c}_{Yb})$ for $c_{Yb}=40\%$. The shape of the curve clearly reflects the presence of three temperature regimes, I, II and III, in Figure 7, related to the temperature-dependent mobility of the dopants. For low temperatures (regime I), say $T^{*}<0.15$, the threshold time τ is longer than the total time of MC simulation (30,000 MCS) because the dopant atoms are practically still. In the second temperature regime (T* = 0.15–0.22), the mobility of the dopants increases, promoting migration in time and decay of large clusters. As a result, a strong (nearly vertical in Figure 12) decrease in the threshold time τ is observed. Finally, in the high-temperature regime III (T* > 0.25), the active migration of the dopants makes the threshold time *τ* short (less than 1000 MCS) and weakly (compared to regime II) dependent on the temperature.

The phase diagram in Figure 12 separates two physically distinct areas in the space of variables (T*, *t*). The left-hand part (marked in yellow) is characterized by large clusters of dopants that promote the cluster-mediated EM. On the contrary, the right-hand part (marked in green) does not support large-distance EM. The crossover between the two regimes may occur due to an increase in the temperature, for fixed time t (horizontal line), or due to an increase in time t, for fixed temperature (vertical line).

#### 3.2.3. Two Types of Dopant Ions

**Figure 13:**
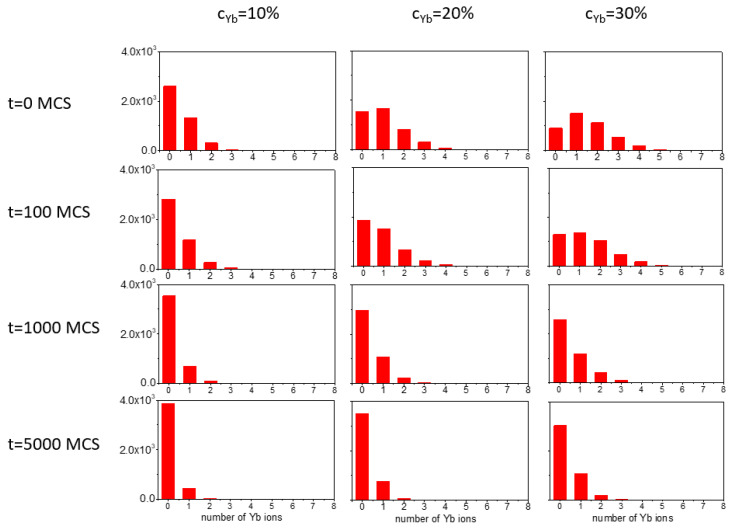
Total number *N*(*k*) of Er ions which have *k* (*k* = 0, …, 8) Yb ions as nearest neighbors, calculated for a few concentrations of Yb ions, and at chosen stages of dopant ion diffusion from the core into the shell. Calculations performed for $c_{Er}=5\%$.

The model can be easily generalized to study the diffusion of two kinds of dopant ions. In particular, the neighborhood relation between Yb and Er ions, crucial for an efficient UC, can be investigated. It is defined in the same way as for Yb-Yb ions: the Er-Yb distance should be below 0.4 nm. The number of Yb ions which are nearest neighbors of an Er ion is a random variable that takes values *k* = 0, …, 8. We have studied the distribution of this random variable by calculating the total number *N*(*k*) of Er ions for each value of k. The concentration of Er ions was 5%, corresponding to about $N_{r}\approx 4300$ Er ions. Figure 13 shows the plots of those distributions for three concentrations (10%, 20% and 30%) of Yb ions in the initial stage in the core, calculated at various times: 0, 100, 1000 and 5000 MCS. In each case, the sum of N(k) over *k* was equal to $N_{r}$, which enables the direct comparison of the distributions. We point out that ratio *N*(*k*)$/N_{r}$ is the probability that the random variable has the value *k*. In general, the numbers *N*(*k*) (*k* = 1, …, 8) decay during the process of diffusion, while *N*(0) increases. An analysis of the distribution for $c_{Yb}=30\%$ indicates that at time t=1000 MCS, it is similar to that for $c_{Yb}=10\%$ at time t=100 MCS. We put forward the hypothesis which states that the distributions for higher concentrations of YB atoms correspond to the distributions for lower concentrations in earlier stages of the diffusion, indicating a kind of scaling relation. A detailed quantitative analysis of such a correspondence goes beyond the scope of this paper.

## 4. Discussion and Conclusions

This paper constitutes a theory-based (MC) continuation of experimental studies and preliminary MC analysis of upconversion in NaYF4:Yb^3+^, Er^3+^ nanocrystals recently reported in Ref. [9]. It is oriented toward the characterization of the temperature-dependent processes related to the clusterization of Yb ions, hypothetically responsible for EM as a percolation-like driven phenomenon [9]. The paper offers a mixture of methodology (generalized MC model), theory (anomalous diffusion, inverse percolation) and potential predictions for experiments.

The MC model generalizes the previous one [9] by accounting for an ion–atom exchange-driven diffusion of dopant Yb/Er ions.

The emerging physical picture is as follows: The dopants undergo a weak subdiffusion in a wide interval of temperatures, interpreted in terms of an obstacle-driven random walk. The role of an obstacle is played by an empty lattice site (i.e., without host Y atom) which is a neighbor for an Yb ion. In spite of the fact that the distribution of the obstacles is not an effect of independent sampling, the weak subdiffusion effect can be surprisingly well reproduced in terms of a toy model of random walk in a simple cubic lattice with independently sampled obstacles. The decrease in efficiency of a UC with MC time is then a result of an “inverse percolation” where large clusters of Yb ions decay in time as the result of a subdiffusion of the dopants. This, in turn, leads to a decrease in the length scale for the EM mechanism, hypothetically responsible for the high efficiency of a UC [9].

The main predictions, which have the character of “no go” conditions, are related to an interplay between an elevated temperature $T^{*}$, Yb ion concentration $c_{Yb}$ and time t (MCS) in the context of the spatial structure/distribution/neighboring relations between Yb ions. The latter can be analyzed either in terms of average nearest distances $\left\langle d_{min}\right\rangle$ or the distribution of the sizes of Yb clusters. The results of the former approach are summarized in Figure 8, Figure 9 and Figure 10. The favorable conditions for EM require that $\left\langle d_{min}\right\rangle$ be comparable to the distance between Yb ions for which the transfer probability cannot be neglected. The data from Figure 11c suggest a “no go” condition:

(4) $$\left\langle d_{min}\right\rangle >(6-7)\;Å$$

Then, there is a relation between temperature, concentration and time which characterizes an efficient EM. For example, for $T^{*}=0.3$ the part of the phase diagram in variables t-$c_{Yb,}$ marked in blue, light blue and light green (Figure 10b), corresponds to an efficient EM. The second approach, oriented toward an analysis of the interplay between the same parameters, uses the concept of a critical threshold time τ (Figure 12) directly related to the percolation of Yb ions. This time, the “no go” condition reads

(5) t>τ.

For example, for $T^{*}=0.2$ and $c_{Yb}=40\%$, an efficient EM is present for *t* < 5000 MCS.

To avoid misinterpretation, let us make an important methodological comment. The phase diagrams of interest depend on scaled temperature and MC time. In the former case, the phase diagrams can be easily re-drawn using real temperature T given the value of potential barrier U. On the other hand, fixing the relation between units of MC and real times, necessary for a real-time interpretation of MC kinetics, constitutes a challenge. Two approaches are possible. The first one requires an advanced quantum chemistry modeling of the over-barrier transitions (at different temperatures) between Yb and Y atoms, in order to find the statistical distribution of transition times. Once known, the migration could be studied again by a classical MC approach, resulting in the relation between the two units of time. The second approach requires a direct comparison of the results of MC modeling with real experiments (it turned out to be successful in our previous study of Surface Relief Gratings [25]). In the present case, the experiments are much more demanding and should be carried out at elevated temperatures. To the best of our knowledge, at present, no such experimental data are available.

Finally, preliminary data may suggest an existence of a scaling relation for the number of Yb ions—the neighbors of Er ions, formulated in terms of concentration $c_{Yb}$ and time MCS; see Figure 13 and the related discussion. If present, it would be free of the problem of the relation between real and MC times—it would be formulated in terms of a ratio of times, which is independent of a unit of time. We believe that a systematic study of this topic can offer direct predictions for real experiments.

## Data Availability

The original contributions presented in the study are included in the article, further inquiries can be directed to the corresponding author.
